# The Effects of Musical Training on Child Development: a Randomized Trial of *El Sistema* in Venezuela

**DOI:** 10.1007/s11121-016-0727-3

**Published:** 2016-11-28

**Authors:** Xiomara Alemán, Suzanne Duryea, Nancy G. Guerra, Patrick J. McEwan, Rodrigo Muñoz, Marco Stampini, Ariel A. Williamson

**Affiliations:** 1 Social Protection and Health Division, Inter-American Development Bank, Washington, DC, USA; 2 Social Sector, Inter-American Development Bank, 1300 New York Ave., NW, Washington, DC, 20577 USA; 3 School of Social Ecology, University of California Irvine, Irvine, CA USA; 4 Department of Economics, Wellesley College, Wellesley, MA USA; 5 Sistemas Integrales, Santiago, Chile; 6 Department of Psychological and Brain Sciences, University of Delaware, Newark, DE USA

**Keywords:** Child development, Executive function, Latin America, Music, Violence exposure

## Abstract

Many studies have explored the links between music and children’s outcomes; however, study designs have not been sufficiently rigorous to support causal findings. This study aims to assess the effects of a large-scale music program on children’s developmental functioning in the context of high rates of exposure to violence. The paper describes the results of an experimental evaluation of Venezuela’s National System of Youth and Children’s Orchestras. The curriculum of the program, better known as “El Sistema,” emphasizes social interactions through group instruction and group performances. The randomized control trial was conducted in 16 music centers between May 2012 and November 2013. In total, 2914 children ages 6–14 participated in the experiment, with approximately half receiving an offer of admission to the program in September 2012 and half in September 2013. The treatment group children participated for one semester more than the control group children. After 1 year, full-sample ITT estimates indicate improved self-control (by 0.10 standard deviations) and reduced behavioral difficulties (by 0.08 standard deviations), both significant at 10% after controlling for multiple hypothesis testing. There were no full-sample effects on other domains. Sub-sample effects are larger among (1) children with less-educated mothers and (2) boys, especially those exposed to violence at baseline. In the latter subgroup, we find lower levels of aggressive behavior. We find that the program improved self-control and reduced behavioral difficulties, with the effects concentrated among subgroups of vulnerable children. The results suggest the importance of devising mechanisms to target resources to the most vulnerable children.

Trial registration: https://clinicaltrials.gov/ct2/show/NCT02369315

**Electronic supplementary material:**

The online version of this article (doi:10.1007/s11121-016-0727-3) contains supplementary material, which is available to authorized users.

## Introduction

The Fundación Musical Simón Bolívar (FMSB) manages a Venezuelan network of *núcleos* (music centers), collectively known as *El Sistema*. Children and youth receive instrumental and choral training in classical music but also in traditional and popular genres. Training includes group and individual practice and regular group performances. Notwithstanding its potential effects on musical ability and appreciation, *El Sistema* emphasizes the importance of holistic child development and social inclusion (Abreu 2009). The instructional model has been internationally praised (Majno 2012; Wakin 2012) and replicated (El Sistema 2015) but subjected to little empirical study.

A vast literature reports associations between musical training and a variety of positive child developmental outcomes (e.g., Hallam 2010), with some studies showing that music experiences are beneficial for violence-exposed populations (e.g., Garrido et al. 2015). However, estimates largely rely on non-random variation in exposure to musical training, which may confound training effects with those of correlated and unobserved variables such as parent income or motivation. A small number of studies randomly assign exposure to musical training, usually focusing on cognitive ability or academic achievement outcomes. Thirty-six weeks of music training improved the general intelligence of Canadian 6 year-olds (Schellenberg 2004). Portuguese third-graders had improved reading abilities but not intelligence after 24 weeks of musical training (Moreno et al. 2009). Four weeks of computer-based listening activities improved verbal (but not spatial) ability of preschool children and performance on a go/no-go task of executive function (Moreno et al. 2011). Boston preschoolers exposed to 6 weeks of parent-accompanied music enrichment showed no differences in vocabulary or numerical discrimination skills relative to a control group (Mehr et al. 2013). A meta-analysis of 19 music intervention studies for children ages 3–12 years, which included a few experiments, found increased visual-spatial skills compared to control conditions (Hetland 2000).

Even less experimental research exists on outcomes beyond intelligence and academic achievement. In a South Korean experiment, 15 weeks of music training improved self-esteem and reduced aggressive behavior among highly aggressive 10–12 year-olds (Choi et al. 2010). Quasi-experimental evaluations of school-based music lessons found impacts on self-esteem in Australia (Rickard et al. 2013) and on school engagement in Finland (Eerola and Eerola 2014).

This study aims to evaluate the effects of a large-scale music program on child functioning in the context of high rates of violence exposure. The study makes four contributions to the literature on musical training and child outcomes. First, it presents the only experimental evidence on the effects of musical training in a developing country with high rates of violence. Second, it is the only experimental evaluation in any country of a scaled-up, government-implemented intervention. Third, it uses a considerably larger sample than prior experiments, as we randomly assigned 2529 guardians (with 2914 children) to early or delayed admission in 16 orchestra centers. Fourth, we measure a wider set of child outcomes than prior research, including self-regulation, behavior, prosocial skills and connections, and cognitive skills.

Study outcomes are based on a theory of change developed through consultation with FMSB administrators, site observations, and the extant literature. Although childhood music participation may contribute to long-term outcomes, such as secondary school graduation and workforce engagement, such outcomes are beyond the scope of this evaluation. Our theory of change thus specifies intermediate processes (short-term outcomes) that have been associated with long-term outcomes and are likely to be affected by short-term participation in *El Sistema*.

We hypothesized that short-term participation in orchestras or choruses may foster positive change in four child functioning domains: self-regulatory skills, behavior, prosocial skills and connections, and cognitive skills. Participation may increase self-regulation skills, or the modulation of emotion and behavior, as it requires dedicated practice as well as turn-taking, patience, and careful monitoring one’s performance to synchronize playing and singing with others (McPherson and Renwick 2001). The collaborative nature of participation in an orchestra or chorus as well as the increased demands for self-regulation suggests that the experience may also increase prosocial behaviors and reduce negative conduct. It may also discourage individual risk-taking (such as playing out of sequence) and reward collective action. Music-making may also foster social bonding, group cohesion, and shared goals (Eerola and Eerola 2014; Kirschner and Tomasello 2010), which could, in turn, increase prosocial connections or engagement with peers and family. Finally, although the program was not designed with an explicit goal of improving cognitive skills, we hypothesized that short-term participation could improve working memory, visual-spatial skills, and processing speed, as these cognitive skills have been associated with musical training (Hetland 2000; Kraus et al. 2014; Schellenberg 2004).

We conducted pre-specified moderation analyses of program outcomes according to several socio-demographic variables. Given that research indicates that the child functioning domains included in our theory of change may be more or less malleable during different developmental periods (e.g., Berger 2011; Skoe and Kraus 2013), we examined program effects by age. As social inclusion is particularly important in contexts of economic inequality and exposure to violence, we additionally examined whether program effects varied by maternal education, a proxy for economic disadvantage, and by violence exposure for male and female subgroups. Violence exposure has deleterious effects on development and has been shown to disproportionately impact disadvantaged youth (Fowler et al. 2009). Rates of youth violence and homicide in Venezuela are among the highest worldwide (Munyo 2013; World Health Organization 2014). We anticipated that disadvantaged and/or violence-exposed youth would benefit the most from orchestra or chorus participation, as the program provides a free, developmental opportunity that includes adult supervision in a safe and accessible setting. Such opportunities are rare or are costly for disadvantaged youth or for those living in high-violence contexts (Roffman et al. 2001). Given that young males in Latin America are at increased risk for both being victimized and perpetrating violence (Munyo 2013), we examined violence exposure by gender in relation to program outcomes, as Venezuelan boys in particular may show increased benefit from structured, supervised engagement in a prosocial activity.

## Method

### Study Design and Experimental Sample

**Fig. 1:**
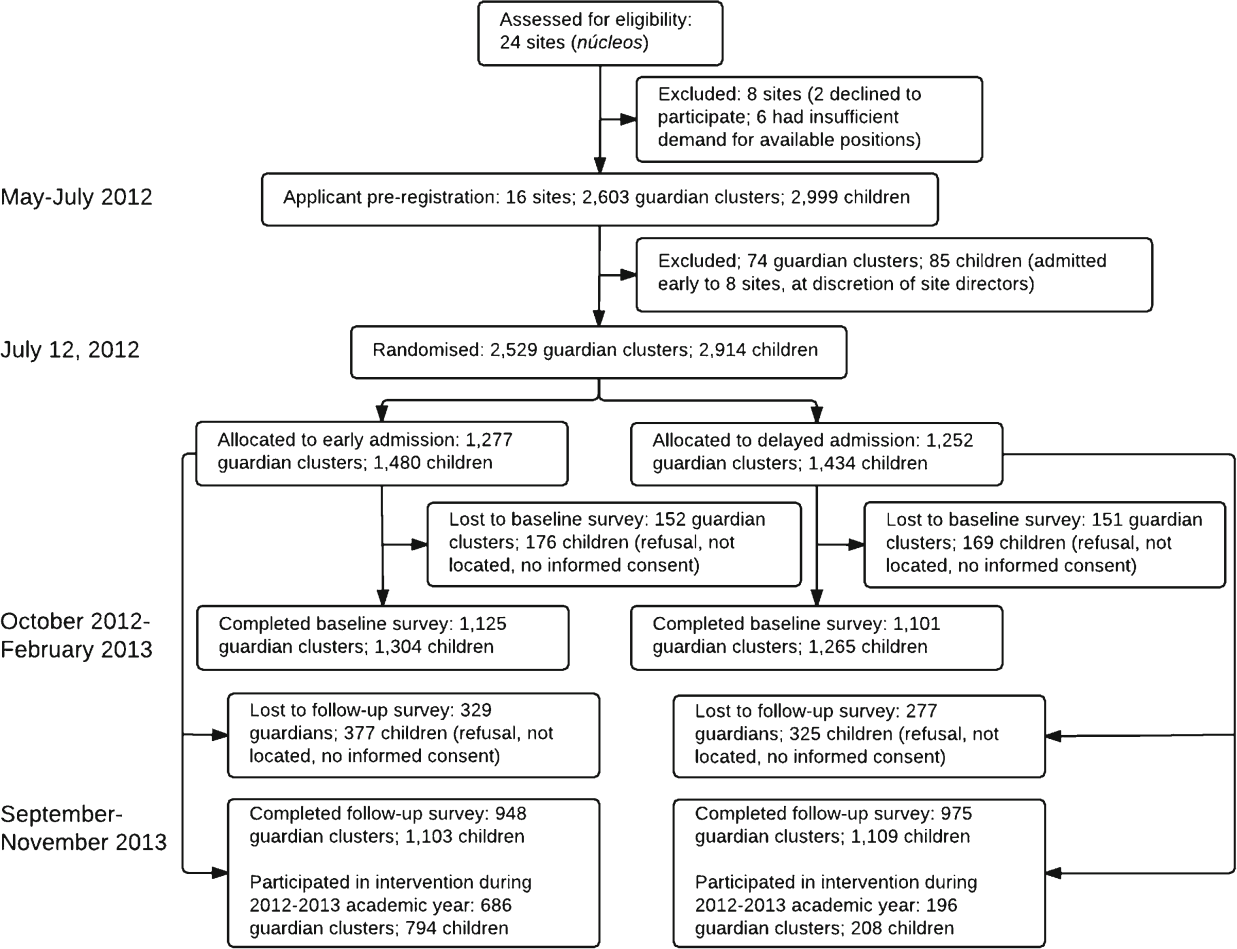
Trial profile

We initially assessed 24 music centers—the experimental sites—for potential inclusion in the experiment (see Fig. 1). In consultation with FMSB administrators, the sites were chosen because of likely excess demand by families in the 2012–2013 academic year and their dispersion across five states: Aragua, Bolívar, the Capital District (Caracas), Lara, and Miranda. Two sites were excluded because their directors declined to follow the experimental protocol, and six were excluded because of insufficient demand. In the remaining 16 sites, directors agreed to participate in the experiment and received training in the experimental protocol.

We conducted a cluster-randomized, controlled trial in the 16 music centers between May 2012 and November 2013. Under normal circumstances, the music centers accept written applications from adult guardians on behalf of one or more children. All children of a particular guardian are admitted on a rolling basis until (and after) classes begin in September. As a condition of participating in the experiment, directors agreed to accept written applications from guardians between May 7 and July 8, 2012 (without informing guardians of admission decisions). Children were eligible to apply if they would be 6 to 14 years old on September 1, 2012. Sites received applications from 2603 guardians on behalf of 2999 children. By prior agreement, each site director could award early admission to a small number of applicants (no more than 5% of positions). In eight sites, 85 children (of 74 guardians) were admitted thusly.

Approximately half of the remaining 2529 guardians (representing 2914 children) were randomly offered early admission in September 2012, with the rest offered admission in September 2013. This constitutes the experimental sample. Baseline and follow-up data were collected on their outcomes, as further described in a later section.

### Randomization and Masking

We randomized admissions to the guardians of children (the experimental clusters), rather than children. This maintained FMSB’s policy of jointly admitting siblings. It also reduced the likelihood of spillover effects between treated and untreated children within a household. On July 12, 2012, we assigned each guardian a random number between 0 and 1, drawn from a continuous uniform distribution. Within each site, guardians and their applicants were allocated to the treatment group (early admission in September 2012) in ascending order of the random number until the number of positions was exhausted. Remaining guardians and children were allocated to the control group (delayed admission in September 2013). The median site allocated 50% of its applicants to the treatment group (with a range of 39 to 67%).

We prepared identically formatted rosters of each site’s treatment and control groups. Site directors were instructed to contact guardians during July and August using a consistent script to provide the early or delayed admission offer. Guardians in the treatment group were not obligated to accept the offer of early admission, and guardians in both groups were not prevented from seeking admission to a non-experimental site. Given the nature of the intervention, it was not possible to prevent guardians and children from learning their treatment status, although treatment status was not revealed to interviewers during baseline or follow-up data collection.

We calculated a minimum detectable effect size (MDES) of 0.106 standard deviations in the randomized sample. This assumes two-tailed hypothesis tests with *α* = 0.05, power of 80%, an intracluster correlation (ICC) of 0.3 (recalling that guardians are the experimental clusters), fixed site effects, and balanced allocation to the treatment and control groups (Schochet 2005). Even with an ICC of 0.6, the MDES is 0.109. The MDES is 0.094 when *α* = 0.1.

### Intervention

Each site includes at least one orchestra and one choir. Sites are guided by a national curriculum (or “sequence”) that specifies compositions and arrangements of increasing complexity, although site directors can modify it at their discretion. During their initial year of participation, school-aged children typically receive instruction in both an instrument (usually the recorder and/or a percussion instrument) and in choral singing. In subsequent years, children select a string, wind, or percussion instrument. Teacher-led musical instruction typically occurs several times per week. The instruction may take place in a full ensemble, within instrument sections, or during individual lessons. Advanced students may additionally provide instruction to less advanced peers. All children, even beginners, are included in public, community performances. Children attend performances of their peers and, in some cases, of regional or national orchestras composed of advanced students. Musical instruction and instruments are free.

### Data Collection

During the initial application period mentioned above, guardians provided written responses to a few demographic and socioeconomic questions. We additionally collected data from guardians and children on two further occasions. First, we conducted a baseline survey of outcome measures between October 2012 and February 2013 (the median survey date was November 19). Surveys were completed in households by children and adult caregivers (usually mothers). Trained enumerators blinded to treatment and control status used laptops and a data entry tool during household interviews. Second, we conducted a similarly structured follow-up survey between September and November 2013 (the median survey date was October 3).

### Outcome Measures

We measured 26 primary outcome variables within the four domains of self-regulatory skills, behaviors, prosocial skills and connections, and cognitive skills. Self-regulation variables include self- and guardian-reported questionnaires as well as computerized games measuring future orientation (delay discount), response inhibition (go/no-go), attention functioning (Flanker task), and planning skills (Tower of London). Regarding behaviors, we focused on self- and guardian-reported measures of broad prosocial behavior, difficulties (Strengths and Difficulties Questionnaire) and aggression, with a risk-taking task (risky driving game). Prosocial skills and connections included scale measures of self-esteem, empathy, and school and family engagement. Cognitive skills included working memory (a digit recall), processing speed (a symbol search), and visual-spatial reasoning. Appendix Table 6 provides information about measure scoring methodology, baseline internal consistency reliability, and references.

### Program Moderators

Child age, gender, and maternal education were collected in the application form. Violence exposure (Pynoos et al. 1998) was collected in the baseline survey. Violence-exposed children responded affirmatively to at least one of the following: “in the city where you live, have you (1) been hit, shot or threatened? (2) seen someone get shot or killed? (3) seen a dead body (except for a funeral)? or (4) learned about the violent death or injury of a loved one?”

### Statistical Analysis

Each outcome includes difference-in-differences estimate of the intention-to-treat (ITT). We used a long-format dataset, in which the number of observations is equal to the number of valid observations on the outcome in the baseline and follow-up surveys, excluding observations missing either a baseline or follow-up measure. We estimated the following regression:

$$ {O}_{ijt}={\beta}_0+{\beta}_1{\mathrm{Post}}_t+{\beta}_2{\mathrm{Treatment}}_j*{\mathrm{Post}}_t+{\delta}_{ij}+{\varepsilon}_{ijt} $$

where ${O}_{ijt}$ is the outcome of child *i* with guardian *j* at time *t*. ${\mathrm{Treatment}}_j$ indicates whether the children of guardian *j* were ever assigned to the treatment group (versus control), while ${\mathrm{Post}}_t$ indicates follow-up observations (versus baseline). The ${\delta}_{ij}$ are fixed effects, or separate intercepts, for each child. (The fixed effects for music centers are absorbed by the child fixed effects and cannot be separately estimated.) The coefficient on the interaction term, ${\beta}_2$, is the ITT effect, that is, the effect of being offered the treatment. Robust standard errors are clustered by guardians. (All reported *p* values are similarly adjusted for clustering.)

Due to the large number of hypothesis tests, we control the *k* familywise error rate (*k*-FWE), or the probability of making *k* or more false rejections. While we follow the Romano-Wolf procedure (Romano et al. 2008; Romano and Wolf 2005), we do not apply the traditional familywise error rate *k*-FWE of *k* = 1, given that this has been shown to be too conservative (Delattre and Roquain 2015). We have set *k* = *h*/2 where *h* is the number of outcomes within a domain (when *h*/2 is not an integer, *k* is rounded down; Guo et al. 2014). There is no doubt a tradeoff between setting the screen for false discoveries too conservatively or too low. In addition to reporting standard *p* values, we report statistical significant at 10% post-adjustment. The adjustment was implemented through a Stata 13.0 bootstrap procedure which establishes an empirical distribution of adjusted critical *t* values. A Matlab algorithm uses this distribution to determine the level of significance according to the Romano-Wolf procedure.

## Results

### Sample Description

Table 1 reports means of demographic and socioeconomic characteristics of households and children in the treatment and control groups. Guardians reported the variables at the time of application from May to July 2012. The table reports adjusted treatment-control differences that control for site-specific dummy variables because the probability of treatment was not equal across sites. As expected, the treatment-control differences in child and household variables are small and not statistically different from zero. Table 2 further shows means for the 26 outcome measures collected during the baseline survey. Each variable is standardized to a z-score, using the baseline mean and standard deviation (follow-up outcomes are standardized using the same values). None of the differences are larger than 10% of a standard deviation.

**Table 1:** Baseline demographic and socioeconomic characteristics

	Treatment group: early admission (*n* = 1480)	Control group: delayed admission (*n* = 1434)	Adjusted difference	*p* value
Child age on September 1, 2012; mean (SD)	9.5 (2.2)	9.5 (2.2)	0.02	0.763
Female	1474 (54%)	1409 (52%)	2.1%	0.255
Child’s household has				
Computer	1464 (84%)	1400 (85%)	0.1%	0.958
Internet	1455 (70%)	1396 (73%)	−0.9%	0.605
Cable television	1461 (79%)	1398 (81%)	−2.3%	0.161
Washing machine	1467 (95%)	1399 (96%)	−0.09%	0.915
Water filter	1461 (55%)	1390 (54%)	1.8%	0.368
Microwave	1461 (69%)	1393 (69%)	0.4%	0.845
Telephone (landline)	1443 (86%)	1391 (85%)	1.1%	0.403
Mother lives with child	1418 (97%)	1348 (96%)	0.3%	0.722
Mother has ≥1 year of tertiary schooling	1416 (54%)	1338 (57%)	−0.8%	0.693
Father lives with child	1298 (76%)	1280 (77%)	−1.5%	0.412
Father has ≥1 year of tertiary schooling	1285 (45%)	1278 (48%)	−2.4%	0.265

All the data were collected from a written survey completed by guardians at the time of application. Values indicate the variable’s sample size (percentage of “yes” responses in parentheses), except for child age. Sample sizes for specific variables are less than the number of randomized students (in the first row) because of survey non-response. Each adjusted difference is calculated from a regression of the row variable on a dummy variable indicating an admission offer in September 2012, as well as *núcleo* fixed effects; the *p* value reflects an adjustment for clustering by guardian

**Table 2:** Definitions of outcome measures and baseline means

Variable (expected sign of the estimate)	Sub-domain	Denominator (individual)		Adjusted difference	*p* value
		Intervention	Control		
		(*n* = 1307)	(*n* = 1268)		
Self-regulatory skills					
Self-control (+)	Self-control	−0.023	0.023	−0.066	0.104
Self-control—guardian (+)	Self-control	−0.037	0.037	−0.083	0.049
Delay discount (+)	Future orientation	−0.050	0.054	−0.084	0.152
Go/no-go-commission (−)	Response inhibition	−0.023	0.024	−0.021	0.618
Flanker, interference score (−)	Attention	−0.030	0.030	−0.075	0.116
Tower of London (−)	Planning skills	−0.018	0.020	−0.041	0.526
Behaviors					
Prosocial behavior (+)	Prosocial behavior	0.008	−0.008	0.020	0.630
Prosocial behavior—guardian (+)	Prosocial behavior	−0.034	0.034	−0.077	0.062
Aggressive behavior (−)	Aggressive behavior	−0.003	0.003	−0.004	0.917
Aggression propensity (−)	Aggression propensity	−0.004	0.004	0.001	0.984
Aggression—guardian (−)	Aggressive behavior	0.022	−0.023	0.060	0.150
Risky driving (−)	Propensity risk-taking	−0.023	0.023	−0.036	0.413
Difficulties (−)	Behavioral difficulties	0.034	−0.035	0.079	0.049
Difficulties—guardian (−)	Behavioral difficulties	0.032	−0.033	0.064	0.122
Interpersonal functioning—guardian (+)	Other related skills	−0.043	0.045	−0.071	0.100
Prosocial skills and connections					
Empathy (+)	Empathy	0.003	−0.003	0.028	0.489
Self-esteem (+)	Self-esteem	−0.016	0.016	−0.023	0.570
Family involvement—guardian (+)	Family engagement	−0.022	0.023	−0.029	0.509
School functioning—guardian (+)	School engagement	−0.007	0.008	−0.013	0.745
Affective strengths—guardian (+)	Affective skills	−0.014	0.014	−0.021	0.622
Career strengths—guardian (+)	Career orientation	−0.018	0.019	−0.037	0.385
Intrapersonal strengths—guardian (+)	Self-related skills	−0.026	0.026	−0.054	0.212
Cognitive skills					
Score forward (+)	Working memory	0.019	−0.019	0.039	0.373
Score backward (+)	Working memory	0.023	−0.023	0.056	0.204
Raven (+)	Visual-spatial skills	−0.023	0.023	−0.034	0.429
Symbol search (+)	Processing speed	−0.006	0.006	−0.020	0.627

All data were collected during the baseline survey. Each adjusted difference is calculated from a regression of the row variable on a dummy variable indicating an admission offer in September 2012, as well as *núcleo* fixed effects; the *p* value reflects an adjustment for clustering by guardian. The sign in the first column indicates the expected direction of the effect

How representative is the experimental sample of the population of similarly aged Venezuelan children in 2012? To partially assess this, we compared the experimental sample to a representative sample from the *Encuesta de Hogares por Muestreo*, collected in the first half of 2012. Among 6 to 14 year-olds in the five states represented in the experiment, 46.5% reside in a household with an income per capita below a poverty line of US$4 per day (or 678 Bs.F. per month). Using a logit specification, we regressed a dummy variable indicating poverty on the child and household variables in Table 1. We used parameter estimates and application form data to predict each experimental child’s probability of residing in a poor household. The sample mean (16.7%) is an estimate of the poverty rate in the experimental sample (Tarozzi and Deaton 2009). We conclude that experimental children are less poor, on average, than all 6 to 14 year-olds residing in the same states. Data limitations prevent us from assessing whether the experimental sample is representative of all applicants to FMSB music centers.

### Attrition

Figure 1 shows participant flow throughout the study. At baseline, 88.1% of treatment group children and 88.2% of controls completed the survey. At follow-up, 74.5% of the treatment group and 77.3% of the control group completed the survey. To examine any systematic differences in attrition by experimental group, Table 3 reports means of demographic and socioeconomic variables from the application form (as in Table 1), but limited to the sample of children who answered at least one question in both the baseline and follow-up surveys. The differences between treatment and control participants are small and not statistically different from zero. Given the possibility of imbalance in unobserved variables, our preferred estimates include child fixed effects that control for any time-invariant unobserved variables.

**Table 3:** Baseline demographic and socioeconomic characteristics for children who answered one question in both the baseline and follow-up surveys

	Treatment group: early admission (*n* = 1044)	Control group: delayed admission (*n* = 1065)	Adjusted difference	*p* value
Child age on September 1, 2012; mean (SD)	9.5 (2.2)	9.4 (2.1)	0.11	0.224
Female	1044 (54%)	1065 (53%)	1.4%	0.533
Child’s household has				
Computer	1037 (84%)	1062 (84%)	1.03%	0.542
Internet	1031 (69%)	1059 (72%)	−0.04%	0.983
Cable television	1034 (78%)	1060 (81%)	−2.57%	0.173
Washing machine	1040 (95%)	1061 (95%)	0.38%	0.700
Water filter	1035 (53%)	1053 (54%)	0.34%	0.889
Microwave	1036 (68%)	1056 (69%)	0.72%	0.753
Telephone (landline)	1024 (85%)	1054 (86%)	−0.18%	0.906
Mother lives with child	1006 (97%)	1025 (96%)	0.87%	0.356
Mother has ≥1 year of tertiary schooling	1006 (54%)	1017 (57%)	−0.99%	0.689
Father lives with child	918 (75%)	978 (78%)	−2.73%	0.210
Father has ≥1 year of tertiary schooling	906 (43%)	975 (47%)	−2.95%	0.249

All data were collected from a written survey completed by guardians at the time of application. Values indicate the variable’s sample size (percentage of “yes” responses in parentheses), except for child age. The sample is defined by all randomized children who responded at least one question in the baseline and the follow-up survey. Sample sizes for specific variables are less than the maximum number of children who responded at least one question in both rounds of survey (in the first row) because of survey non-response. Each adjusted difference is calculated from a regression of the row variable on a dummy variable indicating an admission offer in September 2012, as well as *núcleo* fixed effects; the *p* value reflects an adjustment for clustering by guardian

Table 1 in the supplemental online appendix further compares treatment and control group response rates for each of the 26 outcome variables, conditional on participation. Response is defined as completing a scale or task, and a child is defined as participating if she has non-missing data for both rounds. Response was nearly universal for the scale measures (over 98%), as for these outcomes it was easier to ensure complete answers by repeating a question when necessary. Task outcomes had lower response rates, ranging from 77 to 89%. The lowest response rate was for the Tower of London task, due to the fact that the cumulative test stopped after failure to complete two consecutive trials, so that the difficulty level of the child was not exceeded. This table shows that response rates, conditional on participation, were not statistically significant across treatment and control groups except for the case of one task-based instrument. As this is not among the indicators for which we find an impact, we are reassured that neither attrition nor non-response poses a threat to the internal validity of our analysis.

### Uptake and Implementation

The treatment period was characterized by important political events, including presidential elections on October 7, 2012, and the death of President Hugo Chavez on March 5, 2013. A retrospective qualitative survey collected from directors of the music centers in October 2015 inquired about implementation challenges in recent school years. Only one of the 16 directors reported that implementation in the 2012–2013 school year was temporarily disrupted by school closures related to election activities. Seven directors reported that implementation in 2012–2013 was normal or better compared to other years, and four reported that implementation was worse because of problems with crowding. At worst, interruptions would reduce the dosage of the intervention and dilute the impacts measured. In no case, however, did implementation challenges threaten the internal validity of the evaluation design, as treatment was assigned randomly at the guardian level, and there is no reason to believe that political events may have differentially affected the treatment and control group.

Guardian and child enrollment and attendance were voluntary. In the treatment and control groups, respectively, 69 and 15% of children participated in a music center during the first semester (September to December 2012); 58 and 14% participated during the second semester (January to June 2013); and 56 and 11% participated during both semesters. On average, treatment group children participated 0.98 semesters more than control group children (*p* < 0.001), controlling for site-specific fixed effects and clustering standard errors by guardians. The estimates are based on those who completed the follow-up survey and provided retrospective participation data. Consistent with the design of the experiment, members of the treatment group were not required to enroll, while members of the control group were not prohibited from enrolling in a music center not included in the experiment. The ITT approach addresses crossover by comparing children who were offered early or delayed admission.

The data suggest that music centers offered instruction consistent with *El Sistema* guidelines. Among first-semester participants, 35% received instruction 5 or more days per week and 47% between 2 and 4 days per week (39 and 45% among second-semester participants). During the first semester, 63% received choral training; 67% played an instrument (a recorder, in 6 of 10 cases); and 40% did both. During the second semester, 57% received choral training, 73% played an instrument, and 37% did both. The majority of instruction in the first year occurred in large-group sessions. In two semesters, respectively, only 15 and 18% of participants received section-specific instruction, while 16 and 17% received individual lessons. About half of participants gave a public performance to parents or the public.

### Impacts

Table 4 reports ITT estimates in the full sample of applicants. Two outcomes are statistically significant at 10% after controlling the *k*-FWE (as described above). The offer of early admission to a site increases child-reported self-control by 0.10 standard deviations, compared with delayed admission. It reduced child-reported behavioral difficulties by 0.08 standard deviations. There were no significant effects found for outcomes in other domains.

**Table 4:** ITT estimates in full sample

Variable (expected sign of the estimate)	Denominator (individual)				
	Intervention	Control	ITT effect size (90% CI)		*p* value
	N obs	N obs			
Self-regulatory skills					
Self-control (+)	1027	1051	0.095	(0.013 to 0.177)	0.056^†^
Self-control—guardian (+)	1026	1040	0.039	(−0.036 to 0.113)	0.392
Delay discount (+)	477	446	0.031	(−0.103 to 0.164)	0.705
Go/no-go-commission (−)	853	854	0.037	(−0.057 to 0.131)	0.518
Flanker, interference score (−)	547	561	0.102	(−0.021 to 0.225)	0.171
Tower of London (−)	415	387	0.064	(−0.061 to 0.188)	0.401
Behaviors					
Prosocial behavior (+)	1025	1050	−0.017	(−0.104 to 0.070)	0.748
Prosocial behavior (SDQ)—guardian (+)	1030	1048	0.062	(−0.022 to 0.146)	0.227
Aggressive behavior (−)	1025	1048	−0.015	(−0.108 to 0.079)	0.794
Aggression propensity (−)	1024	1049	−0.011	(−0.091 to 0.068)	0.816
Aggression—guardian (−)	1029	1049	−0.014	(−0.098 to 0.070)	0.781
Risky driving (−)	854	891	0.017	(−0.070 to 0.103)	0.752
Difficulties (−)	1025	1048	−0.081	(−0.160 to −0.003)	0.088^†^
Difficulties—guardian (−)	1029	1048	0.026	(−0.045 to 0.097)	0.547
Interpersonal functioning—guardian (+)	1027	1048	−0.014	(−0.089 to 0.061)	0.762
Prosocial skills and connections					
Empathy (+)	1024	1049	0.007	(−0.073 to 0.086)	0.892
Self-esteem (+)	1027	1051	0.026	(−0.058 to 0.111)	0.61
Family involvement—guardian (+)	1028	1048	−0.063	(−0.152 to 0.025)	0.24
School functioning—guardian (+)	1027	1048	−0.04	(−0.116 to 0.035)	0.38
Affective strengths—guardian (+)	1028	1049	−0.03	(−0.115 to 0.055)	0.565
Career strengths—guardian (+)	1028	1049	0.037	(−0.046 to 0.120)	0.464
Intrapersonal strengths—guardian (+)	1025	1048	−0.035	(−0.119 to 0.049)	0.495
Cognitive skills					
Score forward (+)	904	921	−0.087	(−0.175 to 0.001)	0.106
Score backward (+)	901	915	−0.074	(−0.165 to 0.017)	0.183
Raven (+)	894	921	−0.022	(−0.100 to 0.056)	0.646
Symbol search (+)	917	927	0.024	(−0.060 to 0.108)	0.634

^†^Remains significant for at least 10% level of significance after controlling the *k*-familywise error rate (see text). The ITT estimate is based on the statistical model described in the text

Table 5 reports ITT estimates for moderation analyses within maternal education, gender-by-violence, and age subgroups. It reports estimates that remain statistically significant at 10% after controlling the *k*-FWE (full results are available online). The effect sizes for children with less-educated mothers are approximately 50% higher for self-control and behavioral difficulties than the full-sample estimates, but there were not significant results in these outcomes for children with more-educated mothers. In the latter group, there were also two unexpectedly negative effects on guardian-reported measures of prosocial skills and connections.

**Table 5:** ITT estimates in subgroups

		Denominator (individual)		ITT effect size (90% CI)		*p* value
		Intervention	Control			
		N obs	N obs			
Mother’s education						
Mother without college	Self-regulatory skills					
	Self-control (+)	455	424	0.177	(0.057 to 0.298)	0.016
	Behaviors					
	Difficulties (−)	455	423	−0.134	(−0.253 to −0.016)	0.062
Mother with college	Prosocial skills and connections					
	Family involvement—guardian (+)	535	575	−0.209	(−0.320 to −0.098)	0.002
	Intrapersonal strengths—guardian (+)	533	574	−0.142	(−0.248 to −0.035)	0.028
Gender and violence						
Boys exposed to violence	Behaviors					
	Aggressive behavior (−)	220	223	−0.242	(−0.450 to −0.034)	0.056
	Difficulties (−)	219	224	−0.253	(−0.415 to −0.092)	0.01
	Self-regulatory skills					
	Self-control (+)	220	224	0.205	(0.039 to 0.370)	0.042
Boys not exposed to violence	Self-regulatory skills					
	Self-control (+)	252	266	0.180	(0.022 to 0.337)	0.061
Girls exposed to violence	Prosocial skills and connections					
	Empathy (+)	213	265	−0.179	(−0.336 to −0.023)	0.059
Girls not exposed to violence	Cognitive skills					
	Score backward (+)	300	256	−0.251	(−0.403 to −0.099)	0.007
	Behaviors					
	Prosocial behavior (+)	341	296	−0.167	(−0.316 to −0.017)	0.068
Age						
Younger (aged 6 to 9)	Self-regulatory skills					
	Self-control (+)	544	583	0.134	(0.021 to 0.248)	0.052
Older (aged 10 to 14)	Self-regulatory skills					
	Go/no-go-commission (−)	416	390	0.168	(0.049 to 0.287)	0.02

The table only reports estimates that are statistically significant at 10% after controlling the *k*-familywise error rate (see text). All results are reported in the online appendix

Overall, 46% of males and 42% of females were exposed to violence. The effect sizes for child-reported self-control and behavioral difficulties are more than doubled among boys exposed to violence. An offer of early admission also reduced aggressive behavior in this subgroup by 24% of a standard deviation. The results for girls are less consistent with the theory of change. We find unexpectedly negative effects on empathy (among girls exposed to violence) and on working memory and prosocial behavior (among girls not exposed to violence). The full-sample effect on self-control is observed among younger children (6 to 9 year-olds) but not older children (10 to 14). This finding is consistent with previous research indicating that executive functions and self-regulation skills in particular are more malleable at younger ages (Berger 2011). Finally, there is an unexpectedly negative effect on the go/no-go task among older children.

## Discussion

After 1 year, the early-admission group had higher self-control and fewer behavioral difficulties, based on child reports. Larger effects were found for children with less-educated mothers, which may reflect the ability of more-educated mothers to finance alternative activities. The effects were concentrated among boys, especially those exposed to violence. The latter group also showed reductions in self-reported aggressive behavior. It bears emphasis that these are ITT estimates of the effect of offering the opportunity to enroll, rather than the effect on those actually treated. On average, the early-admission group actually attended about one semester more than the delayed-admission group.

We did not find any full-sample effects on cognitive skills—adding to the mixed findings from wealthier countries (Mehr et al. 2013; Moreno et al. 2009, 2011; Schellenberg 2004)—or on prosocial skills and connections. Unexpectedly, we found few effects for girls overall, with some unexpected decreases in different skill domains. While it could be that males and females engage in different aspects of the program in different ways, further study of *El Sistema* and the Venezuelan context is necessary to better understand these results.

The findings suggest that exposure to *El Sistema* might serve an important role as a preventive strategy to promote positive outcomes among disadvantaged children. The subgroup results are especially relevant given research showing that, relative to their female and higher-income peers, male youth are at increased risk for poor developmental outcomes when exposed to disadvantaged or high-violence contexts (Anderson 2008; Moffitt et al. 2011). That *El Sistema* is particularly effective for vulnerable males is promising, especially as many interventions have been found to be relatively less effective for this group or even to impose adverse effects (Kling et al. 2005; Osypuk et al. 2012; Rodríguez-Planas 2012). While it is possible that group music participation could mitigate the effects of violence exposure for males in particular, experimental studies of this gender effect and the potential benefits of music programs on violence-exposed populations are needed (e.g., Garrido et al. 2015).

Nonetheless, this study highlights the challenges of targeting interventions towards vulnerable groups of children in the context of a voluntary social program. As noted above, just above half of the early-admission group participated in a music center for two semesters. In light of these results, it may be desirable to consider additional targeting and retention mechanisms beyond free tuition and instruments (e.g., travel vouchers, scholarships, or other inducements).

Our lack of findings in cognitive and prosocial skills and connections could be due to the short duration of this evaluation, as changes in these domains may take longer than 1 year to emerge. For example, Kraus et al. (2014) found that changes in neural development took place after 2, but not 1, years of music exposure, suggesting that program duration is important to explore in subsequent studies of *El Sistema*. A benefit of continued study—and a limitation of the present results—is that many children were only exposed to introductory training in a single instrument (the recorder) or none at all. After the first year, children select a single string, wind, or percussion instrument and are exposed to individual and section training (in addition to group training), which could also facilitate positive change in these skill domains.

Although program impacts were concentrated in a few outcomes, these are increasingly identified as critical for individual wellbeing. The long-term economic returns to socio-emotional skills and behaviors can be as large if not larger than the returns to cognitive skills (Cunha and Heckman 2007, 2010). For example, Daly et al. (2015) found that self-control measured at age 11 is predictive of subsequent unemployment at older ages. This is plausibly because the skills that allow children to control their emotions and behavior during school age are closely related to skills used to secure and maintain good jobs and healthy relationships.

### Limitations and Future Directions

There are some limitations of this study that have important implications for future research. Findings are limited to self- and guardian-reported outcomes, which may introduce bias associated with scale measures. Future research should utilize additional reporters (e.g., music directors, peers, or school teachers). In context of longer-run impacts, it is also necessary to examine possible fadeout of impacts and whether cognitive impacts emerge after a longer time frame. Although the evaluation was of a fully scaled program, the generalizability of these results is potentially hampered by a focus on a modest number of music centers. To facilitate experimental assignment, it was limited to over-subscribed music centers which may have implicitly favored better known and/or higher-quality sites. The sample did not affect the internal validity of the results, and assessing the effect on external validity would require additional information on the other music centers. Finally, although we examined program moderators, “how” characteristics such as gender and exposure to violence contribute to program outcomes are unknown; as such, further longitudinal and qualitative research is needed.

Despite these limitations, this study, to our knowledge, presents the only experimental evidence on the effects of musical training in a developing country. Previous research has not adequately addressed causality, as studies have primarily been correlational with the few experiments failing to correct for classroom or school-level clustering. The experiment is also notable for its analysis of a scaled-up, government-implemented musical training intervention. It can thus be considered an effectiveness trial rather than an efficacy trial, perhaps with increased generalizability to the growing body of developing-country policies inspired by *El Sistema*.

### Electronic supplementary material

Below is the link to the electronic supplementary material.

ESM 1 (DOCX 90 kb)

## Appendix

**Table 6:** Measurement information

Measure	Type	Construct/methodology	Reference
Self-regulatory skills			
Self-control (+)	Scale	Self-control; 10 items; *α* = 0.73	Rohrbeck C. A., Azar S.T., & Wagner P. E. (1991). Child self-control rating scale: validation of a child self-report measure. *Journal of Clinical Child Psychology 20*, 179*–*183.
Self-control—guardian (+)	Scale	Self-control; 31 items; *α* = 0.87	Kendall P.C., Wilcox, L.E. (1979). Self-control in children: development of a rating scale. *Journal of Consulting and Clinical Psychology, 47*, 1020–1029.
Delay discount (+)	Game	Future orientation; indifference amount (one day)	Steinberg L., Graham, S., O’Brien, L., Woolard, J. L., Cauffman, E., & Banich, M. (2008). Age differences in future orientation and delay discounting. *Child Development*, 80, 28–44.
Go/no-go average commission error (−)	Game	Response inhibition; average of 2 blocks of 80 trials each	Bezdjian, S., Baker, L.A., Lozano, D.I., Raine, A. (2009). Assessing inattention and impulsivity in children during the Go/NoGo task*. British Journal of Developmental Psychology, 27*, 365–383.
Flanker, interference score (−)	Game	Attention functioning	Eriksen, B.A., Eriksen, C.W. (1974). Effects of noise letters upon the identification of a target letter in a Nonsearch task. *Perception and Psychophysics, 16*,143-149.
Tower of London (−)	Game	Planning skills; sum of total moves over minimum to solve trial	Berg W.K., & Byrd, D. L. (2002). The Tower of London spatial problem-solving task: enhancing clinical and research implementation. *Journal of Clinical and Experimental Neuropsychology, 24*, 586–604.
Behaviors			
Prosocial behavior (SDQ) (+)	Scale	Prosocial behavior; 5 items; *α* = 0.65	Goodman, R. (1997). The Strengths and Difficulties Questionnaire. *Journal of Child Psychology and Psychiatry,40*,133745.
Prosocial behavior (SDQ)—guardian (+)	Scale	Prosocial behavior; 5 items; *α* = 0.60	Goodman (1997)
Aggressive behavior (−)	Scale	Verbal and physical aggression; 5 items; *α* = 0.70	Adapted from: Farrell, A.D., Kung, E. M., White, K.S., & Valois, R.F. (2000). The structure of the self-reported aggression, drug use, and delinquent behaviors during early adolescence. *Journal of Child Clinical Psychology, 29*, 282*–*292.
Aggression propensity (−)	Scale	Physical aggression propensity; 12 items, *α* = 0.87	Chan W., & Henry, D. (2009). *What would make you fight? A measure of motivation for aggression*. Washington, DC, United States: Society for Prevention Research, 2009.
Aggression—guardian (−)	Scale	Verbal and physical aggression; 4 items, *α* = 0.68	European Center for Drug and Drug Addiction. (n.d.) The social and antisocial behavior scale. http://www.emcdda.europa.eu/html.cfm/index3373EN.html (accessed February 5, 2013).
Risky driving (−)	Game	Risk-taking propensity; sum of time elapsed before braking at 7 risky intersections	Steinberg, L., Albert, D., Cauffman, E., Banich, M., Graham, S., & Woolard, J. (2008). Age differences in sensation seeking and impulsivity as indexed by behavior and self-report: evidence for a dual systems model. *Developmental Psychology, 44*, 1764–1778.
Difficulties (−)	Scale	Difficult behaviors, 20 items, *α* = 0.79	Goodman, 1997
Difficulties—guardian (−)	Scale	Difficult behaviors, 20 items, *α* = 0.78	Goodman, 1997
Interpersonal functioning—guardian (+)	Scale	Other-related skills; 15 items; *α* = 0.84	Epstein, M.H., & Sharma, J. (2004). *Behavioral and Emotional Rating Scale-2: A Strengths-Based Approach to Assessment*. Austin, TX: PRO-ED Inc.
Prosocial skills and connections			
Empathy (+)	Scale	Empathy; 10 items, *α* = 0.93	Bryant, B. K. (1982). An index of empathy for children and adolescents. *Child Development*, 53, 413–425.
Self-esteem (+)	Scale	Self-esteem; 5 items, *α* = 0.57	Rosenberg, M. (1965). *Society and the adolescent self-image*. Princeton, NJ: University Press.
Family involvement—guardian (+)	Scale	Family engagement;10 items; *α* = 0.64	Epstein & Sharma, 2004
School functioning—guardian (+)	Scale	School engagement; 9 items; *α* = 0.79	Epstein & Sharma, 2004
Affective strengths—guardian (+)	Scale	Affective expression; 7 items; *α* = 0.71	Epstein & Sharma, 2004
Career strengths—guardian (+)	Scale	Career orientation; 5 items; *α* = 0.65	Epstein & Sharma, 2004
Intrapersonal strengths—guardian(+)	Scale	Self-related skills; 11 items; *α* = 0.76	Epstein & Sharma, 2004
Cognitive skills			
Digit recall forward (+)	Game	Working memory; sum of correct	Luciana, M., Collins, P.F., Olson, E.A., & Schissel, A. M. (2009). The development of planning skills and associations with self-reported inattention and impulsivity. *Developmental Neuropsychology, 34*, 461–475.
Digit recall backward (+)	Game	Working memory; sum of correct	Luciana et. al., 2009.
Raven (+)	Game	Visual-spatial reasoning; sum of correct	Raven, J. C. (1956). *Guide to the Coloured Progressive Matrices Sets A, Ab, B. 1st ed*. London, UK: Lewis.
Symbol search (+)	Game	Processing speed; number correct minus incorrect in 120 s	Adapted from: Carlozzi, N.E., Tulsky, N. D., Beaumont, J. L., Weintraub, S., Conway, K., Gershon, R. C. (2014). NIH toolbox cognitive battery (NIHTB-CB): The NIHTB pattern comparison processing speed test. *Journal of the International Neuropsychological Society; 20*(6), 630–641.
