# Supplementary material for: The Effects of Musical Training on Child Development: a Randomized Trial of El Sistema in Venezuela
Source: Prev Sci. 2016 Nov 28;18(7):865–78. doi: 10.1007/s11121-016-0727-3 (PMC5602103; doi:10.1007/s11121-016-0727-3)
Supplement: Supplementary file 1 — (DOCX 90 kb) [file 11121_2016_727_MOESM1_ESM.docx]

**Supplementary appendix**

Supplement to: The effects of musical training on child development:

a randomized trial of *El Sistema* in Venezuela

Xiomara Alemán, MS; Suzanne Duryea, PhD; Nancy G. Guerra, PhD; Patrick J. McEwan, PhD; Rodrigo Muñoz, MS; Marco Stampini, PhD; Ariel A. Williamson, MA

Author Affiliations:

Social Protection and Health Division, Inter-American Development Bank, Washington DC, USA (Aleman, Stampini); Social Sector, Inter-American Development Bank, Washington DC, USA (Duryea); Department of Psychological and Brain Sciences, University of Delaware, Newark DE, USA (Guerra, Williamson); Department of Economics, Wellesley College, Wellesley MA, USA (McEwan); Sistemas Integrales, Santiago, Chile (Muñoz).

Corresponding author:

Suzanne Duryea, PhD

Inter-American Development Bank

1300 New York Avenue NW, Stop E0601, Washington DC 20577, USA;

(202) 623-3589;

suzanned@iadb.org

**Table of Contents**

[Response](#_Supplementary_Table_1.) Rates in Both Survey Rounds by Instrument ……………………………….…………………….……..1

[Supplementary Tables of Intent to Treat Results by Sub-groups](#_Supplementary_Tables_of) ………………………………………………….…4

[Gender and violence](#_Gender_and_violence)……………………………………………….……………………………………….……….4

[Mother’s education](#_Mother´s_education)………………………………………………………………………………….………..……..8

[Age](#_Age)……………………………………………………………………………………………………………….…10

**Supplementary Table 1. *Response Rates by Instrument - Sample of children who answered at least one question in both the baseline and follow-up surveys***

|  | **Treatment group: early admission**  **(n=1044)** | **Control group: delayed admission**  **(n=1065)** | **Adjusted difference** | **p-value** |
| --- | --- | --- | --- | --- |
| **Scales** |  |  |  |  |
| Self-control | 98.6% | 98.6% | -0.10% | 0.8587 |
| Self-control - Guardian | 99.1% | 98.2% | 0.65% | 0.2592 |
| Prosocial behavior | 98.4% | 98.5% | -0.26% | 0.6818 |
| Prosocial behavior (SDQ) - Guardian | 99.6% | 99.1% | 0.40% | 0.3806 |
| Aggressive behavior | 98.4% | 98.3% | -0.05% | 0.9429 |
| Aggression propensity | 98.3% | 98.4% | -0.25% | 0.6991 |
| Aggression - Guardian | 99.5% | 99.1% | 0.29% | 0.5341 |
| Difficulties | 98.4% | 98.3% | -0.01% | 0.9931 |
| Difficulties - Guardian | 99.5% | 99.1% | 0.29% | 0.5408 |
| Interpersonal functioning - Guardian | 99.3% | 99.0% | 0.19% | 0.7043 |
| Empathy | 98.3% | 98.4% | -0.25% | 0.6989 |
| Self-esteem | 98.6% | 98.6% | -0.10% | 0.8587 |
| Family involvement - Guardian | 99.4% | 99.0% | 0.29% | 0.5652 |
| School functioning - Guardian | 99.3% | 99.0% | 0.22% | 0.6692 |
| Affective strengths - Guardian | 99.4% | 99.1% | 0.19% | 0.6952 |
| Career strengths - Guardian | 99.3% | 99.1% | 0.08% | 0.8688 |
| Intrapersonal strengths - Guardian | 99.1% | 99.0% | -0.04% | 0.9391 |
|  |  |  |  |  |
| **Games** |  |  |  |  |
| Delay discount | 89.5% | 85.8% | 4.02% | 0.0676 |
| Go/no-go-Commission | 81.9% | 80.2% | 0.17% | 0.9230 |
| Flanker, interference score | 83.8% | 84.0% | -1.48% | 0.3805 |
| Tower of London | 77.6% | 74.3% | 3.96% | 0.1479 |
| Risky driving | 81.9% | 83.7% | -2.48% | 0.1495 |
| Score forward | 86.7% | 86.5% | 0.05% | 0.9765 |
| Score backward | 86.4% | 85.9% | 0.31% | 0.8450 |
| Raven | 85.7% | 86.5% | -1.53% | 0.3340 |
| Symbol search | 87.9% | 87.1% | -0.23% | 0.8788 |
|  |  |  |  |  |

Data were collected from survey rounds. The sample is defined by all randomized children who responded at least one question in the baseline and the follow-up surveys. Sample for Delay Discount and Tower of London was restricted to valid participants: children 10 years old or older (n= 974 obs.; Treatment= 495 and Control= 479). Values indicated in the 2^nd^ and 3^rd^ columns represent the "participation rates" or the percentage of children from whom we collected outcome information in both rounds of survey (variable outcomes for these children were not “missing”) for the treatment and control groups, respectively. Each adjusted difference is calculated from a regression of the row variable’s participation rate on a dummy variable indicating an admission offer in September 2012, as well as *núcleo* fixed effects; the p-value reflects an adjustment for clustering by guardian.

## Supplementary Tables of Intent to Treat (ITT) Results by Sub-groups

###

| Supplementary Table 2. *ITT estimates in subgroups: Boys not exposed to violence* | | | | |  |
| --- | --- | --- | --- | --- | --- |
|  | Denominator(individual) | |  |  |  |
|  | Intervention (N obs) | Control (N obs) | ITT effect size (90% CI) | | P value |
| **Self-regulatory Skills** |  |  |  |  |  |
| Self-control (+) | 252 | 266 | 0.180* | (0.022 to 0.337) | 0.061 † |
| Self-control - Guardian (+) | 253 | 262 | 0.050 | (-0.102 to 0.202) | 0.589 |
| Delay discount (+) | 97 | 87 | 0.047 | (-0.267 to 0.361) | 0.803 |
| Go/no-go-Commission (-) | 202 | 216 | 0.005 | (-0.171 to 0.180) | 0.965 |
| Flanker, interference score (-) | 140 | 153 | 0.175 | (-0.074 to 0.423) | 0.247 |
| Tower of London (-) | 84 | 75 | 0.119 | (-0.201 to 0.439) | 0.538 |
| **Behaviors** |  |  |  |  |  |
| Prosocial behavior (+) | 252 | 265 | 0.067 | (-0.120 to 0.254) | 0.555 |
| Prosocial behavior (SDQ) Guardian (+) | 254 | 265 | 0.081 | (-0.090 to 0.251) | 0.436 |
| Aggressive behavior (-) | 251 | 264 | 0.130 | (-0.065 to 0.325) | 0.274 |
| Aggression propensity (-) | 250 | 264 | -0.021 | (-0.190 to 0.147) | 0.834 |
| Aggression - Guardian (-) | 254 | 266 | 0.024 | (-0.152 to 0.200) | 0.823 |
| Risky driving (-) | 202 | 218 | 0.116 | (-0.058 to 0.290) | 0.272 |
| Difficulties (-) | 252 | 265 | -0.008 | (-0.160 to 0.145) | 0.932 |
| Difficulties - Guardian (-) | 254 | 266 | 0.071 | (-0.068 to 0.210) | 0.402 |
| Interpersonal functioning - Guardian (+) | 253 | 266 | 0.082 | (-0.063 to 0.226) | 0.352 |
| **ProSocial Skills and Connections** |  |  |  |  |  |
| Empathy (+) | 250 | 265 | 0.069 | (-0.090 to 0.228) | 0.477 |
| Self-esteem (+) | 252 | 266 | 0.088 | (-0.088 to 0.264) | 0.409 |
| Family involvement - Guardian (+) | 254 | 266 | -0.011 | (-0.178 to 0.157) | 0.914 |
| School functioning - Guardian (+) | 252 | 266 | 0.033 | (-0.116 to 0.182) | 0.712 |
| Affective strengths - Guardian (+) | 254 | 266 | 0.119 | (-0.048 to 0.286) | 0.240 |
| Career strengths - Guardian (+) | 254 | 266 | 0.087 | (-0.082 to 0.256) | 0.399 |
| Intrapersonal strengths - Guardian (+) | 252 | 265 | -0.030 | (-0.193 to 0.132) | 0.760 |
| **Cognitive Skills** |  |  |  |  |  |
| Score forward (+) | 219 | 236 | -0.077 | (-0.254 to 0.099) | 0.471 |
| Score backward (+) | 218 | 234 | -0.017 | (-0.184 to 0.150) | 0.868 |
| Raven (+) | 213 | 227 | -0.013 | (-0.167 to 0.141) | 0.892 |
| Symbol search (+) | 219 | 232 | 0.123 | (-0.040 to 0.285) | 0.214 |
|  |  |  |  |  |  |
| † Remains significant for at least 10% level of significance after controlling the k-Family wise Error Rate (see text). The ITT estimate is based on the statistical model described in the text. | | | | | |

| Supplementary Table 3. *ITT estimates in subgroups: Boys exposed to violence* | | | | |  |
| --- | --- | --- | --- | --- | --- |
|  | Denominator(individual) | |  |  |  |
|  | Intervention (N obs) | Control (N obs) | ITT effect size (90% CI) | | P value |
| **Self-regulatory Skills** |  |  |  |  |  |
| Self-control (+) | 220 | 224 | 0.205** | (0.039 to 0.370) | 0.042 † |
| Self-control - Guardian (+) | 220 | 222 | 0.152 | (-0.007 to 0.311) | 0.115 |
| Delay discount (+) | 129 | 122 | -0.196 | (-0.423 to 0.030) | 0.154 |
| Go/no-go-Commission (-) | 192 | 186 | -0.025 | (-0.220 to 0.170) | 0.833 |
| Flanker, interference score (-) | 118 | 129 | 0.048 | (-0.225 to 0.321) | 0.772 |
| Tower of London (-) | 112 | 108 | -0.020 | (-0.256 to 0.217) | 0.891 |
| **Behaviors** |  |  |  |  |  |
| Prosocial behavior (+) | 220 | 224 | 0.089 | (-0.104 to 0.281) | 0.448 |
| Prosocial behavior (SDQ) Guardian (+) | 220 | 222 | 0.068 | (-0.115 to 0.251) | 0.542 |
| Aggressive behavior (-) | 220 | 223 | -0.242* | (-0.450 to -0.034) | 0.056 † |
| Aggression propensity (-) | 220 | 224 | 0.005 | (-0.154 to 0.164) | 0.958 |
| Aggression - Guardian (-) | 220 | 222 | -0.011 | (-0.180 to 0.157) | 0.912 |
| Risky driving (-) | 180 | 192 | 0.030 | (-0.142 to 0.202) | 0.773 |
| Difficulties (-) | 219 | 224 | -0.253** | (-0.415 to -0.092) | 0.010 † |
| Difficulties - Guardian (-) | 219 | 222 | -0.099 | (-0.246 to 0.049) | 0.271 |
| Interpersonal functioning - Guardian (+) | 220 | 222 | 0.009 | (-0.150 to 0.168) | 0.926 |
| **ProSocial Skills and Connections** |  |  |  |  |  |
| Empathy (+) | 220 | 224 | 0.005 | (-0.157 to 0.167) | 0.96 |
| Self-esteem (+) | 220 | 224 | -0.081 | (-0.254 to 0.093) | 0.444 |
| Family involvement - Guardian (+) | 219 | 221 | -0.076 | (-0.253 to 0.102) | 0.482 |
| School functioning - Guardian (+) | 220 | 221 | 0.005 | (-0.164 to 0.173) | 0.964 |
| Affective strengths - Guardian (+) | 219 | 222 | -0.073 | (-0.245 to 0.098) | 0.482 |
| Career strengths - Guardian (+) | 220 | 222 | 0.066 | (-0.117 to 0.249) | 0.553 |
| Intrapersonal strengths - Guardian (+) | 218 | 222 | 0.028 | (-0.140 to 0.195) | 0.787 |
| **Cognitive Skills** |  |  |  |  |  |
| Score forward (+) | 198 | 200 | -0.024 | (-0.219 to 0.171) | 0.839 |
| Score backward (+) | 198 | 199 | 0.104 | (-0.099 to 0.308) | 0.398 |
| Raven (+) | 191 | 201 | -0.016 | (-0.187 to 0.156) | 0.880 |
| Symbol search (+) | 203 | 202 | 0.088 | (-0.093 to 0.270) | 0.422 |
|  |  |  |  |  |  |
| † Remains significant for at least 10% level of significance after controlling the k-Family wise Error Rate (see text). The ITT estimate is based on the statistical model described in the text. | | | | | |

| Supplementary Table 4. *ITT estimates in subgroups: Girls not exposed to violence* | | | | |  |
| --- | --- | --- | --- | --- | --- |
|  | Denominator(individual) | |  |  |  |
|  | Intervention (N obs) | Control (N obs) | ITT effect size (90% CI) | | P value |
| **Self-regulatory Skills** |  |  |  |  |  |
| Self-control (+) | 342 | 296 | 0.063 | (-0.082 to 0.208) | 0.476 |
| Self-control - Guardian (+) | 340 | 295 | -0.006 | (-0.125 to 0.113) | 0.937 |
| Delay discount (+) | 140 | 108 | 0.078 | (-0.196 to 0.351) | 0.641 |
| Go/no-go-Commission (-) | 277 | 237 | -0.036 | (-0.199 to 0.127) | 0.717 |
| Flanker, interference score (-) | 184 | 133 | 0.158 | (-0.074 to 0.389) | 0.261 |
| Tower of London (-) | 122 | 94 | 0.168 | (-0.071 to 0.407) | 0.248 |
| **Behaviors** |  |  |  |  |  |
| Prosocial behavior (+) | 341 | 296 | -0.167* | (-0.316 to -0.017) | 0.068 † |
| Prosocial behavior (SDQ) Guardian (+) | 343 | 297 | 0.045 | (-0.102 to 0.192) | 0.616 |
| Aggressive behavior (-) | 341 | 296 | 0.000 | (-0.160 to 0.160) | 0.997 |
| Aggression propensity (-) | 341 | 296 | -0.005 | (-0.133 to 0.122) | 0.947 |
| Aggression - Guardian (-) | 342 | 297 | -0.053 | (-0.202 to 0.096) | 0.559 |
| Risky driving (-) | 282 | 258 | -0.003 | (-0.168 to 0.162) | 0.975 |
| Difficulties (-) | 341 | 294 | -0.059 | (-0.200 to 0.082) | 0.493 |
| Difficulties - Guardian (-) | 343 | 297 | 0.046 | (-0.087 to 0.180) | 0.566 |
| Interpersonal functioning - Guardian (+) | 342 | 296 | -0.044 | (-0.174 to 0.086) | 0.580 |
| **ProSocial Skills and Connections** |  |  |  |  |  |
| Empathy (+) | 341 | 295 | 0.079 | (-0.066 to 0.224) | 0.371 |
| Self-esteem (+) | 342 | 296 | 0.106 | (-0.041 to 0.253) | 0.236 |
| Family involvement - Guardian (+) | 342 | 297 | -0.081 | (-0.234 to 0.071) | 0.379 |
| School functioning - Guardian (+) | 342 | 297 | -0.077 | (-0.202 to 0.048) | 0.312 |
| Affective strengths - Guardian (+) | 342 | 297 | -0.161* | (-0.306 to -0.017) | 0.067 |
| Career strengths - Guardian (+) | 341 | 297 | -0.038 | (-0.176 to 0.099) | 0.645 |
| Intrapersonal strengths - Guardian (+) | 342 | 297 | -0.081 | (-0.228 to 0.066) | 0.364 |
| **Cognitive Skills** |  |  |  |  |  |
| Score forward (+) | 300 | 257 | -0.140 | (-0.296 to 0.016) | 0.139 |
| Score backward (+) | 300 | 256 | -0.251*** | (-0.403 to -0.099) | 0.007 † |
| Raven (+) | 299 | 264 | -0.068 | (-0.206 to 0.071) | 0.422 |
| Symbol search (+) | 304 | 265 | -0.058 | (-0.211 to 0.095) | 0.529 |
|  |  |  |  |  |  |
| † Remains significant for at least 10% level of significance after controlling the k-Family wise Error Rate (see text). The ITT estimate is based on the statistical model described in the text. | | | | | |

| Supplementary Table 5. *ITT estimates in subgroups: Girls exposed to violence* | | | | |  |
| --- | --- | --- | --- | --- | --- |
|  | Denominator(individual) | |  |  |  |
|  | Intervention (N obs) | Control (N obs) | ITT effect size (90% CI) | | P value |
| **Self-regulatory Skills** |  |  |  |  |  |
| Self-control (+) | 213 | 265 | -0.040 | (-0.210 to 0.129) | 0.695 |
| Self-control - Guardian (+) | 213 | 261 | -0.023 | (-0.161 to 0.116) | 0.789 |
| Delay discount (+) | 111 | 129 | 0.223 | (-0.060 to 0.506) | 0.195 |
| Go/no-go-Commission (-) | 182 | 215 | 0.206* | (0.005 to 0.407) | 0.091 |
| Flanker, interference score (-) | 105 | 146 | 0.062 | (-0.187 to 0.310) | 0.682 |
| Tower of London (-) | 97 | 110 | 0.024 | (-0.189 to 0.237) | 0.853 |
| **Behaviors** |  |  |  |  |  |
| Prosocial behavior (+) | 212 | 265 | -0.001 | (-0.163 to 0.162) | 0.994 |
| Prosocial behavior (SDQ) Guardian (+) | 213 | 264 | 0.078 | (-0.085 to 0.240) | 0.431 |
| Aggressive behavior (-) | 213 | 265 | 0.003 | (-0.176 to 0.182) | 0.978 |
| Aggression propensity (-) | 213 | 265 | -0.049 | (-0.220 to 0.122) | 0.638 |
| Aggression - Guardian (-) | 213 | 264 | -0.019 | (-0.179 to 0.142) | 0.849 |
| Risky driving (-) | 190 | 223 | -0.085 | (-0.262 to 0.093) | 0.433 |
| Difficulties (-) | 213 | 265 | -0.041 | (-0.208 to 0.126) | 0.687 |
| Difficulties - Guardian (-) | 213 | 263 | 0.062 | (-0.076 to 0.201) | 0.460 |
| Interpersonal functioning - Guardian (+) | 212 | 264 | -0.092 | (-0.238 to 0.054) | 0.298 |
| **ProSocial Skills and Connections** |  |  |  |  |  |
| Empathy (+) | 213 | 265 | -0.179* | (-0.336 to -0.023) | 0.059 † |
| Self-esteem (+) | 213 | 265 | 0.006 | (-0.165 to 0.178) | 0.952 |
| Family involvement - Guardian (+) | 213 | 264 | -0.061 | (-0.235 to 0.113) | 0.562 |
| School functioning - Guardian (+) | 213 | 264 | -0.111 | (-0.264 to 0.041) | 0.229 |
| Affective strengths - Guardian (+) | 213 | 264 | 0.043 | (-0.125 to 0.212) | 0.672 |
| Career strengths - Guardian (+) | 213 | 264 | 0.084 | (-0.083 to 0.251) | 0.409 |
| Intrapersonal strengths - Guardian (+) | 213 | 264 | -0.021 | (-0.186 to 0.143) | 0.830 |
| **Cognitive Skills** |  |  |  |  |  |
| Score forward (+) | 187 | 228 | -0.089 | (-0.248 to 0.071) | 0.359 |
| Score backward (+) | 185 | 226 | -0.082 | (-0.274 to 0.109) | 0.480 |
| Raven (+) | 191 | 229 | 0.031 | (-0.121 to 0.182) | 0.737 |
| Symbol search (+) | 191 | 228 | -0.019 | (-0.195 to 0.157) | 0.862 |
|  |  |  |  |  |  |
| † Remains significant for at least 10% level of significance after controlling the k-Family wise Error Rate (see text). The ITT estimate is based on the statistical model described in the text. | | | | | |

###

| Supplementary Table 6. *ITT estimates in subgroups: Mother without college* | | | | |  |
| --- | --- | --- | --- | --- | --- |
|  | Denominator(individual) | |  |  |  |
|  | Intervention (N obs) | Control (N obs) | ITT effect size (90% CI) | | P value |
| **Self-regulatory Skills** |  |  |  |  |  |
| Self-control (+) | 455 | 424 | 0.177** | (0.057 to 0.298) | 0.016 † |
| Self-control - Guardian (+) | 455 | 421 | -0.005 | (-0.131 to 0.122) | 0.952 |
| Delay discount (+) | 226 | 188 | 0.153 | (-0.060 to 0.366) | 0.237 |
| Go/no-go-Commission (-) | 376 | 347 | 0.093 | (-0.056 to 0.243) | 0.305 |
| Flanker, interference score (-) | 241 | 224 | 0.128 | (-0.070 to 0.326) | 0.286 |
| Tower of London (-) | 196 | 157 | 0.118 | (-0.072 to 0.307) | 0.307 |
| **Behaviors** |  |  |  |  |  |
| Prosocial behavior (+) | 454 | 424 | -0.043 | (-0.175 to 0.090) | 0.595 |
| Prosocial behavior (SDQ) Guardian (+) | 456 | 425 | 0.106 | (-0.033 to 0.245) | 0.209 |
| Aggressive behavior (-) | 455 | 422 | -0.003 | (-0.155 to 0.150) | 0.977 |
| Aggression propensity (-) | 454 | 423 | 0.018 | (-0.114 to 0.150) | 0.825 |
| Aggression - Guardian (-) | 456 | 425 | 0.039 | (-0.099 to 0.177) | 0.642 |
| Risky driving (-) | 385 | 363 | -0.032 | (-0.165 to 0.101) | 0.690 |
| Difficulties (-) | 455 | 423 | -0.134* | (-0.253 to -0.016) | 0.062 † |
| Difficulties - Guardian (-) | 456 | 424 | 0.077 | (-0.038 to 0.193) | 0.271 |
| Interpersonal functioning - Guardian (+) | 454 | 425 | 0.011 | (-0.113 to 0.135) | 0.881 |
| **ProSocial Skills and Connections** |  |  |  |  |  |
| Empathy (+) | 454 | 423 | 0.003 | (-0.120 to 0.127) | 0.968 |
| Self-esteem (+) | 455 | 424 | 0.055 | (-0.081 to 0.192) | 0.502 |
| Family involvement - Guardian (+) | 456 | 424 | 0.048 | (-0.098 to 0.194) | 0.588 |
| School functioning - Guardian (+) | 455 | 425 | -0.068 | (-0.191 to 0.055) | 0.363 |
| Affective strengths - Guardian (+) | 456 | 425 | 0.049 | (-0.096 to 0.193) | 0.579 |
| Career strengths - Guardian (+) | 456 | 425 | 0.030 | (-0.112 to 0.172) | 0.727 |
| Intrapersonal strengths - Guardian (+) | 455 | 425 | 0.055 | (-0.084 to 0.194) | 0.515 |
| **Cognitive Skills** |  |  |  |  |  |
| Score forward (+) | 399 | 371 | -0.136 | (-0.272 to 0.001) | 0.103 |
| Score backward (+) | 396 | 369 | -0.085 | (-0.228 to 0.058) | 0.328 |
| Raven (+) | 403 | 375 | -0.008 | (-0.135 to 0.118) | 0.915 |
| Symbol search (+) | 410 | 377 | 0.065 | (-0.064 to 0.194) | 0.409 |
|  |  |  |  |  |  |
| † Remains significant for at least 10% level of significance after controlling the k-Family wise Error Rate (see text). The ITT estimate is based on the statistical model described in the text. | | | | | |

| Supplementary Table 7. *ITT estimates in subgroups: Mother with college* | | | | |  |
| --- | --- | --- | --- | --- | --- |
|  | Denominator(individual) | |  |  |  |
|  | Intervention (N obs) | Control (N obs) | ITT effect size (90% CI) | | P value |
| **Self-regulatory Skills** |  |  |  |  |  |
| Self-control (+) | 535 | 578 | 0.039 | (-0.077 to 0.155) | 0.581 |
| Self-control - Guardian (+) | 534 | 570 | 0.067 | (-0.027 to 0.161) | 0.241 |
| Delay discount (+) | 230 | 238 | -0.054 | (-0.228 to 0.121) | 0.613 |
| Go/no-go-Commission (-) | 443 | 470 | -0.029 | (-0.155 to 0.096) | 0.700 |
| Flanker, interference score (-) | 284 | 309 | 0.050 | (-0.113 to 0.214) | 0.612 |
| Tower of London (-) | 203 | 211 | -0.025 | (-0.197 to 0.147) | 0.808 |
| **Behaviors** |  |  |  |  |  |
| Prosocial behavior (+) | 534 | 577 | -0.003 | (-0.122 to 0.116) | 0.968 |
| Prosocial behavior (SDQ) Guardian (+) | 537 | 574 | 0.025 | (-0.080 to 0.131) | 0.693 |
| Aggressive behavior (-) | 533 | 577 | -0.031 | (-0.152 to 0.090) | 0.671 |
| Aggression propensity (-) | 533 | 577 | -0.046 | (-0.146 to 0.054) | 0.449 |
| Aggression - Guardian (-) | 536 | 575 | -0.065 | (-0.172 to 0.041) | 0.313 |
| Risky driving (-) | 438 | 484 | 0.061 | (-0.057 to 0.179) | 0.395 |
| Difficulties (-) | 533 | 576 | -0.064 | (-0.172 to 0.043) | 0.326 |
| Difficulties - Guardian (-) | 536 | 575 | -0.017 | (-0.110 to 0.075) | 0.757 |
| Interpersonal functioning - Guardian (+) | 536 | 574 | -0.038 | (-0.134 to 0.057) | 0.512 |
| **ProSocial Skills and Connections** |  |  |  |  |  |
| Empathy (+) | 533 | 577 | -0.024 | (-0.131 to 0.083) | 0.715 |
| Self-esteem (+) | 535 | 578 | 0 | (-0.111 to 0.111) | 0.996 |
| Family involvement - Guardian (+) | 535 | 575 | -0.209*** | (-0.320 to -0.098) | 0.002 † |
| School functioning - Guardian (+) | 535 | 574 | -0.042 | (-0.140 to 0.055) | 0.477 |
| Affective strengths - Guardian (+) | 535 | 575 | -0.110* | (-0.214 to -0.007) | 0.080 |
| Career strengths - Guardian (+) | 535 | 575 | 0.029 | (-0.074 to 0.131) | 0.648 |
| Intrapersonal strengths - Guardian (+) | 533 | 574 | -0.142** | (-0.248 to -0.035) | 0.028 † |
| **Cognitive Skills** |  |  |  |  |  |
| Score forward (+) | 469 | 505 | -0.033 | (-0.155 to 0.089) | 0.655 |
| Score backward (+) | 469 | 501 | -0.072 | (-0.197 to 0.052) | 0.337 |
| Raven (+) | 459 | 501 | -0.047 | (-0.148 to 0.055) | 0.449 |
| Symbol search (+) | 471 | 506 | 0.010 | (-0.106 to 0.126) | 0.887 |
|  |  |  |  |  |  |
| † Remains significant for at least 10% level of significance after controlling the k-Family wise Error Rate (see text). The ITT estimate is based on the statistical model described in the text. | | | | | |

| Supplementary Table 8. *ITT estimates in subgroups: Younger - age 6 to 9* | | | | |  |
| --- | --- | --- | --- | --- | --- |
|  | Denominator(individual) | |  |  |  |
|  | Intervention (N obs) | Control (N obs) | ITT effect size (90% CI) | | P value |
| **Self-regulatory Skills** |  |  |  |  |  |
| Self-control (+) | 544 | 583 | 0.134* | (0.021 to 0.248) | 0.052 † |
| Self-control - Guardian (+) | 544 | 573 | 0.022 | (-0.071 to 0.115) | 0.699 |
| Delay discount (+) | 35 | 35 | 0.253 | (-0.363 to 0.868) | 0.496 |
| Go/no-go-Commission (-) | 437 | 464 | -0.082 | (-0.221 to 0.057) | 0.332 |
| Flanker, interference score (-) | 275 | 308 | 0.156 | (-0.026 to 0.337) | 0.158 |
| Tower of London (-) | 31 | 31 | -0.279 | (-0.743 to 0.186) | 0.321 |
| **Behaviors** |  |  |  |  |  |
| Prosocial behavior (+) | 542 | 583 | -0.021 | (-0.139 to 0.098) | 0.772 |
| Prosocial behavior (SDQ) Guardian (+) | 545 | 579 | 0.080 | (-0.029 to 0.189) | 0.227 |
| Aggressive behavior (-) | 542 | 583 | 0.048 | (-0.089 to 0.186) | 0.561 |
| Aggression propensity (-) | 541 | 583 | -0.073 | (-0.192 to 0.045) | 0.309 |
| Aggression - Guardian (-) | 545 | 580 | -0.054 | (-0.169 to 0.061) | 0.440 |
| Risky driving (-) | 436 | 479 | 0.046 | (-0.081 to 0.172) | 0.553 |
| Difficulties (-) | 543 | 583 | -0.081 | (-0.190 to 0.027) | 0.217 |
| Difficulties - Guardian (-) | 544 | 580 | 0.040 | (-0.056 to 0.136) | 0.493 |
| Interpersonal functioning - Guardian (+) | 544 | 580 | -0.014 | (-0.112 to 0.085) | 0.819 |
| **ProSocial Skills and Connections** |  |  |  |  |  |
| Empathy (+) | 541 | 582 | -0.022 | (-0.131 to 0.086) | 0.734 |
| Self-esteem (+) | 544 | 583 | 0.076 | (-0.048 to 0.201) | 0.314 |
| Family involvement - Guardian (+) | 544 | 580 | -0.019 | (-0.132 to 0.094) | 0.782 |
| School functioning - Guardian (+) | 544 | 580 | -0.068 | (-0.167 to 0.032) | 0.263 |
| Affective strengths - Guardian (+) | 545 | 580 | -0.027 | (-0.131 to 0.077) | 0.667 |
| Career strengths - Guardian (+) | 544 | 580 | 0.122* | (0.008 to 0.237) | 0.079 |
| Intrapersonal strengths - Guardian (+) | 543 | 579 | -0.029 | (-0.137 to 0.080) | 0.664 |
| **Cognitive Skills** |  |  |  |  |  |
| Score forward (+) | 451 | 492 | -0.109 | (-0.230 to 0.012) | 0.139 |
| Score backward (+) | 450 | 488 | -0.122 | (-0.247 to 0.003) | 0.109 |
| Raven (+) | 459 | 503 | -0.021 | (-0.132 to 0.089) | 0.750 |
| Symbol search (+) | 472 | 510 | -0.020 | (-0.128 to 0.088) | 0.763 |
|  |  |  |  |  |  |
| † Remains significant for at least 10% level of significance after controlling the k-Family wise Error Rate (see text). The ITT estimate is based on the statistical model described in the text. | | | | | |

| Supplementary Table 9. *ITT estimates in subgroups: Older - age 10 to 14* | | | | |  |
| --- | --- | --- | --- | --- | --- |
|  | Denominator(individual) | |  |  |  |
|  | Intervention (N obs) | Control (N obs) | ITT effect size (90% CI) | | P value |
| **Self-regulatory Skills** |  |  |  |  |  |
| Self-control (+) | 483 | 468 | 0.057 | (-0.053 to 0.167) | 0.390 |
| Self-control - Guardian (+) | 482 | 467 | 0.059 | (-0.052 to 0.169) | 0.382 |
| Delay discount (+) | 442 | 411 | 0.013 | (-0.124 to 0.149) | 0.879 |
| Go/no-go-Commission (-) | 416 | 390 | 0.168** | (0.049 to 0.287) | 0.020 † |
| Flanker, interference score (-) | 272 | 253 | 0.043 | (-0.122 to 0.208) | 0.668 |
| Tower of London (-) | 384 | 356 | 0.093 | (-0.036 to 0.221) | 0.235 |
| **Behaviors** |  |  |  |  |  |
| Prosocial behavior (+) | 483 | 467 | -0.002 | (-0.126 to 0.122) | 0.976 |
| Prosocial behavior (SDQ) Guardian (+) | 485 | 469 | 0.043 | (-0.083 to 0.168) | 0.575 |
| Aggressive behavior (-) | 483 | 465 | -0.095 | (-0.212 to 0.022) | 0.182 |
| Aggression propensity (-) | 483 | 466 | 0.056 | (-0.040 to 0.153) | 0.335 |
| Aggression - Guardian (-) | 484 | 469 | 0.035 | (-0.083 to 0.152) | 0.628 |
| Risky driving (-) | 418 | 412 | -0.010 | (-0.125 to 0.105) | 0.884 |
| Difficulties (-) | 482 | 465 | -0.082 | (-0.194 to 0.029) | 0.223 |
| Difficulties - Guardian (-) | 485 | 468 | 0.010 | (-0.094 to 0.114) | 0.875 |
| Interpersonal functioning - Guardian (+) | 483 | 468 | -0.013 | (-0.122 to 0.096) | 0.845 |
| **ProSocial Skills and Connections** |  |  |  |  |  |
| Empathy (+) | 483 | 467 | 0.051 | (-0.059 to 0.162) | 0.444 |
| Self-esteem (+) | 483 | 468 | -0.031 | (-0.140 to 0.077) | 0.633 |
| Family involvement - Guardian (+) | 484 | 468 | -0.115 | (-0.241 to 0.011) | 0.132 |
| School functioning - Guardian (+) | 483 | 468 | -0.009 | (-0.117 to 0.099) | 0.894 |
| Affective strengths - Guardian (+) | 483 | 469 | -0.031 | (-0.161 to 0.099) | 0.693 |
| Career strengths - Guardian (+) | 484 | 469 | -0.061 | (-0.177 to 0.055) | 0.385 |
| Intrapersonal strengths - Guardian (+) | 482 | 469 | -0.042 | (-0.161 to 0.077) | 0.561 |
| **Cognitive Skills** |  |  |  |  |  |
| Score forward (+) | 453 | 429 | -0.060 | (-0.185 to 0.064) | 0.425 |
| Score backward (+) | 451 | 427 | -0.010 | (-0.138 to 0.118) | 0.898 |
| Raven (+) | 435 | 418 | -0.013 | (-0.120 to 0.093) | 0.838 |
| Symbol search (+) | 445 | 417 | 0.071 | (-0.060 to 0.202) | 0.375 |
|  |  |  |  |  |  |
| † Remains significant for at least 10% level of significance after controlling the k-Family wise Error Rate (see text). The ITT estimate is based on the statistical model described in the text. | | | | | |
